# Targeting the dopaminergic midbrain in Alzheimer’s disease: Therapeutic potential of focusing on specific neural circuits rather than single molecular pathways

**DOI:** 10.4103/NRR.NRR-D-25-00925

**Published:** 2026-01-27

**Authors:** Emanuele Claudio Latagliata, Stefano Puglisi-Allegra, Marcello D’Amelio

**Affiliations:** Department of Psychology, International Telematic University Uninettuno, Corso Vittorio Emanuele II, Rome, Italy; Istituto Di Ricovero E Cura a Carattere Scientifico (IRCCS) Neuromed, Via Atinense, Pozzilli, Italy; Department of Experimental Neurosciences, IRCCS Santa Lucia Foundation, Via del Fosso Di Fiorano, Rome, Italy; Department of Medicine and Surgery, Università Campus Bio-Medico Di Roma, Via Alvaro del Portillo, Rome, Italy

Alzheimer’s disease (AD) remains one of the most unyielding challenges in neurology. Its complexity and heterogeneity underscore a critical need to explore additional mechanisms that can be targeted in the early stages of the disease to prevent the worsening of functional decline.

Converging basic-science and emerging clinical evidence now implicates progressive degeneration of the mesocorticolimbic dopamine (DA) system, particularly neurons in the ventral tegmental area (VTA), as a newly recognized event that predicts the onset of memory loss and occurs well before amyloid-β (Aβ) plaque deposition (Sala et al., 2021), as demonstrated in the Tg2576 mouse model of AD (Nobili et al., 2017; Cordella et al., 2018; La Barbera et al., 2022; Spoleti et al., 2024). In this context, our recent work shows that repetitive anodal transcranial direct current stimulation (tDCS) over the prefrontal cortex (**Figure 1**) can boost the firing of the residual VTA dopaminergic neurons, re-establish dopaminergic signaling in VTA projecting areas, and thus ameliorate a wide range of AD hallmarks (De Paolis et al., 2025).

**Figure 1:**
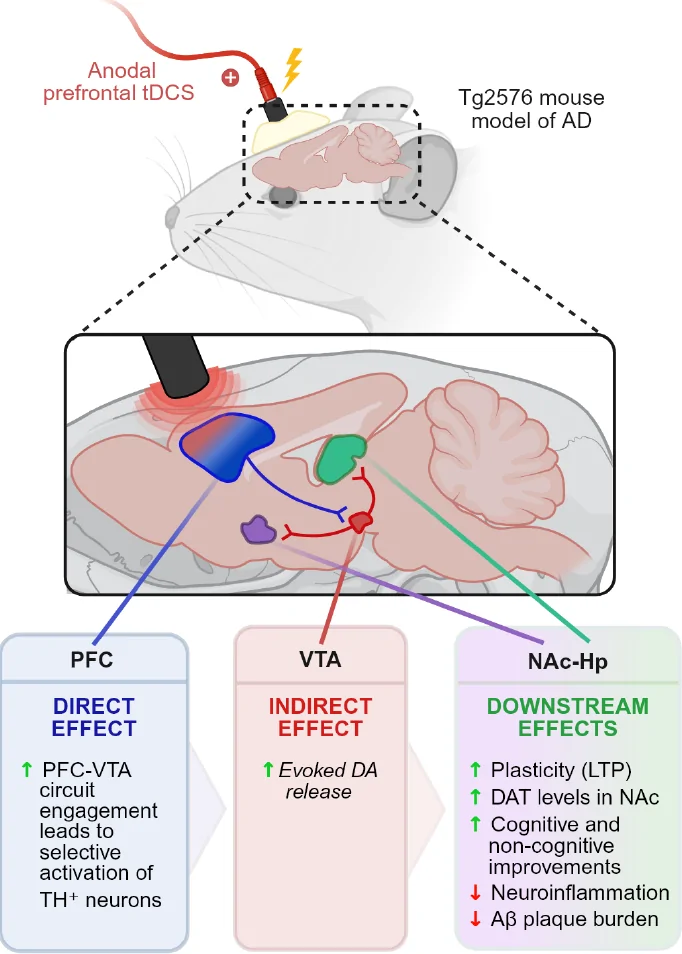
Hypothetical neural mechanisms of tDCS over the PFC in AD. Dopaminergic neurons in the VTA receive excitatory glutamatergic transmission directly and/or indirectly from the PFC. Thus, tDCS stimulation of the PFC, which projects to midbrain dopaminergic neurons, may enhance dopaminergic activity in the VTA. Furthermore, tDCS over the PFC may reorganize the functional connectivity of both the hippocampus and nucleus accumbens via the VTA. The blue line indicates direct and/or indirect projections from the PFC, while the red lines represent dopaminergic projections. Created with BioRender.com. AD: Alzheimer’s disease; Aβ: amyloid-β; DA: dopamine; DAT: dopamine transporter; LTP: long-term potentiation; NAc-Hp: nucleus accumbens-hippocampus; PFC: prefrontal cortex; tDCS: transcranial direct current stimulation; TH: tyrosine hydroxylase; VTA: ventral tegmental area.

These findings argue for a shift from the traditional view of AD as a purely cortico-centric proteinopathy toward a circuit-centric model in which subcortical hubs shape both cognitive and non-cognitive/neuropsychiatric trajectories. Neuroimaging studies in AD patients reveal that VTA volume loss and disrupted connectivity correlate with apathy and depression, symptoms that frequently emerge in prodromal stages, as well as with an acceleration of cognitive decline (Serra et al., 2018; Sala et al., 2021). In Tg2576 mice, prefrontal tDCS restored the dopaminergic tone in the hippocampus and nucleus accumbens, boosted synaptic plasticity, reduced microglial activation and Aβ burden, and even enhanced recognition memory and motivational drive (De Paolis et al., 2025). Such multifaceted effects highlight the therapeutic promise of targeting specific neural circuits rather than single molecular pathways.

**Mechanistic insights:** Prefrontal tDCS is a non-invasive brain stimulation (NIBS) technique, already approved or under investigation for major depression and age-related cognitive deficits because it is safe and inexpensive. In our experiments, c-Fos mapping demonstrated that clinically relevant stimulation parameters selectively activated tyrosine-hydroxylase-positive (TH^+^) VTA neurons without recruiting those of the locus coeruleus. Enhanced DA release–detected upon KCl-induced depolarization–was accompanied by stronger hippocampal long-term potentiation, the electrophysiological cornerstone of declarative memory, and measurable behavioral improvements, despite the ongoing neuronal loss (De Paolis et al., 2025). These findings indicate that tDCS does not directly restore baseline DA levels. Instead, it amplifies the responsiveness of surviving neurons to depolarizing inputs, reinforcing and stabilizing memory and reward circuits during engagement. This mechanism may be especially beneficial in prodromal AD. However, because AD is heterogeneous, clinical responses are expected to vary. Supporting this, functional magnetic resonance imaging studies delineate a subgroup of patients with early VTA disconnection who progress rapidly from mild cognitive impairment to dementia (Serra et al., 2021). Quantitative imaging of VTA structure, connectivity, or ligand uptake could thus guide patient selection and serve as surrogate markers for monitoring, paving the way for precision neuromodulation.

**Evidence from preclinical and human studies:** Mechanistic preclinical studies further show that prefrontal tDCS restores CA3-to-CA1 long-term potentiation, normalizes DA transporter expression in mesolimbic circuits, reduces microglial activation, and lowers Aβ plaque burden (De Paolis et al., 2025)—effects consistent with the ability of DA to mitigate neuroinflammation and disrupt Aβ aggregation (Watamura et al., 2024).

In humans, small randomized studies support feasibility and potential benefit. Acute improvements in recognition memory were observed after anodal stimulation over prefrontal or temporoparietal targets (Ferrucci et al., 2008; Khedr et al., 2014; Im et al., 2019). A 10-session double-blind trial over the left dorsolateral prefrontal cortex showed Mini-Mental State Examination gains and reduced P300 latency, persisting up to 2 months (Khedr et al., 2014). Conversely, a six-session trial over the left temporal cortex did not show benefits, underscoring the importance of montage, dosing, and disease stage (Ferrucci et al., 2008). Notably, a 6-month home-based daily dorsolateral prefrontal cortex protocol in early AD improved Mini-Mental State Examination and Boston Naming scores *versus* sham and attenuated fluorodeoxyglucose-positron emission tomography metabolic decline, highlighting scalability (Im et al., 2019). Ongoing trials are testing caregiver-delivered and combined paradigms with exercise (NCT04855643). Replace across studies with importantly, adverse effects were mild (e.g., tingling, transient scalp burning) and tDCS was well tolerated (Khedr et al., 2014).

**Neural *versus* structural regeneration:** We used neural regeneration to denote functional recovery via synaptic plasticity, network reweighting, and compensatory recruitment, without requiring new neurons. Structural regeneration, by contrast, implies lasting anatomical repair such as neurogenesis, tract-level axonal sprouting, remyelination, or measurable gray-matter gains.

Current tDCS findings in AD and aging align more with neural than structural regeneration. Reported volumetric or microstructural changes are heterogeneous and often small, likely reflecting hemodynamic or glial modulation rather than neuron replacement. Moreover, adult hippocampal neurogenesis remains debated, cautioning against strong claims of structural repair. Together, these data suggest that tDCS should be framed as a circuit-level modulator that preserves or enhances function in vulnerable networks, while definitive structural regeneration remains unproven. Blinded, multimodal studies are needed to adjudicate this issue.

**A roadmap for clinical translation:** A practical roadmap begins with imaging-based screening for VTA atrophy or hypofunction, followed by detailed phenotyping of memory and motivational disturbances, and implementation of anodal prefrontal tDCS. Ongoing reassessment of biomarkers and cognitive outcomes enables adaptive protocols and integration with adjunctive therapies, such as dopaminergic agents, physical exercise, or structured cognitive training.

***Protocol considerations:*** (1) Intensity: 1.5–2.0 mA (titrated to 2.5–3.0 mA if tolerated); (2) Duration: 20–30 minutes with 15–30-second ramps; (3) Electrodes: 25–35 cm² sponge pads (≤ 0.08 mA/cm²); (4) Induction: 5 sessions/week for 2–4 weeks (10–20 in total); (5) Maintenance: 1–3 sessions/week for 3–6 months; (6) Montage: left dorsolateral prefrontal cortex (F3) or medial prefrontal cortex (PFC) as anode; contralateral supraorbital/deltoid cathode; high-definition tDCS for cortical atrophy.

***Integration:*** Deliver tDCS online with cognitive training or prior to exercise to leverage plasticity windows. For patients on dopaminergic drugs, align sessions with therapeutic “on” periods.

***Personalization and safety:*** Adjust montage and dose based on imaging and symptom severity. Monitor adverse events (scalp erythema, tingling, and headache) systematically. Contraindications include active skin lesions, unstable seizures, or implanted stimulators without clearance.

***Monitoring and long-term assessment:*** Combine magnetic resonance imaging (structural, diffusion), functional magnetic resonance imaging/electroencephalogram (connectivity and plasticity), and magnetic resonance spectroscopy or positron emission tomography where feasible. Baseline, post-induction, and follow-ups every 4–8 weeks are recommended. Home-based delivery should include remote safety check-ins.

**Comparative positioning:** Compared to symptomatic pharmacotherapies, which act globally with modest and transient benefit, tDCS targets circuit plasticity directly and avoids systemic side effects. Relative to disease-modifying Aβ/tau therapies, it is inexpensive, portable, and scalable, with a complementary mechanism. Compared to deep brain stimulation, tDCS is non-invasive, deployable earlier, and feasible for home use, though effect sizes may be smaller and state-dependent. Practically, tDCS can be positioned as: (1) an adjunct to pharmacological regimens; (2) a bridge before invasive neuromodulation; (3) a maintenance tool between clinical visits.

**Future directions and outlook:** To translate these findings, systematic validation is essential. Clinical trials must confirm that feasible protocols can reliably activate deep midbrain regions, and must define optimal dosing schedules to avoid paradoxical sensitization or desensitization. Biomarker-driven stratification is critical to avoid dilution of effects in heterogeneous populations.

Long-term studies should also examine whether repeated stimulation confers durable neuroprotective effects and alters disease trajectory. Combining tDCS with dopaminergic agents or behavioral interventions is a promising strategy (Sárkány et al., 2025).

In summary, mounting evidence positions the mesocorticolimbic DA pathway not as a passive bystander but as a driving force in AD pathology. Non-invasive, circuit-specific neuromodulation of VTA *via* prefrontal tDCS holds promise to slow or even partially reverse both molecular hallmarks and behavioral deficits. The next critical step is to translate these insights into precision-medicine protocols, using imaging biomarkers to stratify patients, optimize stimulation settings, and track circuit-level recovery, to deliver truly personalized interventions that ease AD burden on patients and their families.

Moreover, because dopaminergic dysfunction underlies both AD and Parkinson’s disease, another common neurodegenerative disease worldwide, similar tDCS approaches could be adapted to the nigrostriatal system. By targeting residual *substantia nigra* output and the basal ganglia circuits, NIBS may improve motor and non-motor symptoms and potentially slow the disease progression. This opens new frontiers for DA-focused, non-invasive therapies across neurodegenerative disorders.

*The authors would like to thank Dr. Maria Luisa De Paolis for their assistance with the preparation of Figure.*

*This work was supported by the American Alzheimer’s Association [AARG-18–566270; AARG-21–851219], by the Italian Ministry of Health [Research Grant: RF-2018–12365527], by the Italian Ministry of Universities and Research [Prot. 2020Z73J5A] and by Fondazione Roma (Rome, Italy) (to MDA).*

## References

[R1] Cordella A, Krashia P, Nobili A, Pignataro A, La Barbera L, Viscomi MT, Valzania A, Keller F, Ammassari-Teule M, Mercuri NB, Berretta N, D’Amelio M (2018). Dopamine loss alters the hippocampus-nucleus accumbens synaptic transmission in the Tg2576 mouse model of Alzheimer’s disease. Neurobiol Dis.

[R2] De Paolis ML, Loffredo G, Krashia P, La Barbera L, Nobili A, Cauzzi E, Babicola L, Di Segni M, Coccurello R, Puglisi-Allegra S, Latagliata EC, D’Amelio M (2025). Repetitive prefrontal tDCS activates VTA dopaminergic neurons, resulting in attenuation of Alzheimer’s disease-like deficits in Tg2576 mice. Alzheimers Res Ther.

[R3] Ferrucci R, Mameli F, Guidi I, Mrakic-Sposta S, Vergari M, Marceglia S, Cogiamanian F, Barbieri S, Scarpini E, Priori A (2008). Transcranial direct current stimulation improves recognition memory in Alzheimer disease. Neurology.

[R4] Im JJ, Jeong H, Bikson M, Woods AJ, Unal G, Oh JK, Na S, Park JS, Knotkova H, Song IU, Chung YA (2019). Effects of 6-month at-home transcranial direct current stimulation on cognition and cerebral glucose metabolism in Alzheimer’s disease. Brain Stimul.

[R5] Khedr EM, Gamal NFE, El-Fetoh NA, Khalifa H, Ahmed EM, Ali AM, Noaman M, El-Baki AA, Karim AA (2014). A double-blind randomized clinical trial on the efficacy of cortical direct current stimulation for the treatment of Alzheimer’s disease. Front Aging Neurosci.

[R6] La Barbera L, Nobili A, Cauzzi E, Paoletti I, Federici M, Saba L, Giacomet C, Marino R, Krashia P, Melone M, Keller F, Mercuri NB, Viscomi MT, Conti F, D’Amelio M (2022). Upregulation of Ca2+-binding proteins contributes to VTA dopamine neuron survival in the early phases of Alzheimer’s disease in Tg2576 mice. Mol Neurodegener.

[R7] Nobili A (2017). Dopamine neuronal loss contributes to memory and reward dysfunction in a model of Alzheimer’s disease. Nat Commun.

[R8] Sala A, Caminiti SP, Presotto L, Pilotto A, Liguori C, Chiaravalloti A, Garibotto V, Frisoni GB, D’Amelio M, Paghera B, Schillaci O, Mercuri N, Padovani A, Perani D (2021). *In vivo* human molecular neuroimaging of dopaminergic vulnerability along the Alzheimer’s disease phases. Alzheimers Res Ther.

[R9] Sárkány Z, Damásio J, Macedo-Ribeiro S, Martins PM (2025). Association between the use of levodopa/carbidopa, Alzheimer’s disease biomarkers, and cognitive decline among participants in the National Alzheimer’s Coordinating Center Uniform Data Set. Alzheimers Dement.

[R10] Serra L, D’Amelio M, Di Domenico C, Dipasquale O, Marra C, Mercuri NB, Caltagirone C, Cercignani M, Bozzali M (2018). *In vivo* mapping of brainstem nuclei functional connectivity disruption in Alzheimer’s disease. Neurobiol Aging.

[R11] Serra L, D’Amelio M, Esposito S, Domenico CD, Koch G, Marra C, Mercuri NB, Caltagirone C, Artusi CA, Lopiano L, Cercignani M, Bozzali M (2021). Ventral tegmental area disconnection contributes two years early to correctly classify patients converted to Alzheimer’s disease: implications for treatment. J Alzheimers Dis.

[R12] Spoleti E, La Barbera L, Cauzzi E, De Paolis ML, Saba L, Marino R, Sciamanna G, Di Lazzaro V, Keller F, Nobili A, Krashia P, D’Amelio M (2024). Dopamine neuron degeneration in the ventral tegmental area causes hippocampal hyperexcitability in experimental Alzheimer’s disease. Mol Psychiatry.

[R13] Watamura N, Kakiya N, Fujioka R, Kamano N, Takahashi M, Nilsson P, Saito T, Iwata N, Fujisawa S, Saido TC (2024). The dopaminergic system promotes neprilysin-mediated degradation of amyloid-β in the brain. Sci Signal.

